# Limitations of the S-TOFHLA in measuring poor numeracy: a cross-sectional study

**DOI:** 10.1186/s12889-018-5333-9

**Published:** 2018-03-27

**Authors:** Ashley J. Housten, Lisa M. Lowenstein, Diana S. Hoover, Viola B. Leal, Geetanjali R. Kamath, Robert J. Volk

**Affiliations:** 1 Department of Health Services Research, Division of Cancer Prevention & Population Sciences, The University of Texas MD Anderson Cancer Center, 1515 Holcombe Blvd.Unit 1444, Houston, TX 77030 USA; 2 Department of Health Disparities Research, Division of Cancer Prevention & Population Sciences, The University of Texas MD Anderson Cancer Center, 1515 Holcombe Blvd.Unit 1440, Houston, TX 77030 USA; 3 School of Public Health, The University of Texas Health Science Center at Houston, 1200 Pressler St., Houston, TX 77030 USA

**Keywords:** Health literacy, Numeracy, Decision-making, Health disparities

## Abstract

**Background:**

Although the Short Test of Functional Health Literacy in Adults (S-TOFHLA) is widely used, misidentification of individuals with low health literacy (HL) in specific HL dimensions, like numeracy, is a concern. We examined the degree to which individuals scored as “adequate” HL on the S-TOFHLA would be considered as having low HL by two additional numerical measures.

**Methods:**

English-speaking adults aged 45–75 years were recruited from a large, urban academic medical center and a community foodbank in the United States. Participants completed the S-TOFHLA, the Subjective Numeracy Scale (SNS), and the Graphical Literacy Measure (GL), an objective measure of a person’s ability to interpret numeric information presented graphically. Established cut-points or a median split classified participants and having high and low numeracy.

**Results:**

Participants (*n* = 187), on average were: aged 58 years; 63% female; 70% Black/African American; and 45% had a high school degree or less. Of those who scored “adequate” on the S-TOFHLA, 50% scored low on the SNS and 40% scored low on GL. Correlation between the S-TOFHLA and the SNS Total was moderate (*r* = 0.22, *n* = 186, *p* = 0.01), while correlation between the S-TOFHLA and the GL Total was large (*r* = 0.53, *n* = 187, *p* ≤ 0.01).

**Conclusions:**

Findings suggest that the S-TOFHLA may not capture an individuals’ HL in the dimension of numeracy. Efforts are needed to develop more encompassing and practical strategies for identifying those with low HL for use in research and clinical practice.

**Trial registration:**

NCT02151032 (retrospectively registered: May 30, 2014).

## Background

Health literacy (HL) represents a complex intersection of skills needed to “obtain, process, understand, and communicate about health-related information needed to make informed health decisions.” [1–3] The 2003 National Assessment of Adult Literacy (NAAL), a representative survey of 19,000 adults in the United States (US), found that approximately half of all adults demonstrate HL related difficulties, and over one-third (36%) have basic or below basic HL [4]. Compared to individuals with higher HL, those with limited HL are found to use fewer preventive services (e.g., cancer screening) and are more likely to engage in unhealthy behaviors (e.g., poor medication adherence), resulting in increased risk for hospitalization and diminished health outcomes [5, 6]. Furthermore, estimates suggest that low HL costs the US economy between $106–$238 billion annually, and accounts for between 7%–17% of personal healthcare expenses [7]. Due to the common occurrence of limited HL, and its corresponding social and economic impact on population health, it is a top public health priority [5, 8]. With a recent shift in healthcare practice to prioritize patient involvement in medical decision making, measuring HL in order to evaluate patient abilities, develop patient-centered interventions, and promote patient empowerment in the healthcare setting continues to gain support [8, 9]. Given the importance of HL, our aim was to look at the performance of the most commonly used HL measure, The Short Test of Functional Health Literacy in Adults (S-TOFHLA) [9–11].

HL measures are useful for evaluating and classifying patient abilities so that information can be presented in a way to meet patients’ skills and needs. Yet, existing measures of HL may lack the specificity to accurately assess patients’ ability to comprehend numeric information, providing a limited view of patients’ abilities [8, 9, 12–14]. The comprehensive measurement of HL is challenging within clinical settings because HL includes multiple elements, such as print literacy, speaking and listening (oral and aural literacy), cultural knowledge, social skills, and numeracy [2, 9, 13, 15, 16]. Numeracy, defined as one’s aptitude with probabilities, fractions and ratios [16, 17], is of primary interest among those focused on developing risk communication strategies to promote patient engagement in healthcare decisions [14, 18]. Risk estimates and numerical information designed to depict probabilities, percentages, frequencies and trade-offs are widely used in patient decision support materials such as decision aids, but are often poorly understood even among those with higher HL [8, 9, 13, 18–20]. Objective numeracy measures provide insight into individuals’ ability to understand numerical and quantitative information; yet, individuals may be reluctant to objective test questions (e.g. math test questions, probability test questions) and more amenable to subjective measures (e.g., self-reported comfort with numbers, preference for numerical information), without compromising clinical utility [12, 17, 21]. While there is general consensus about the importance of evaluating HL and its associated dimensions, there is no agreed upon “gold-standard” measure, and there is limited agreement about which dimensions of HL can be measured while maintaining clinical feasibility [9, 22]. Moreover, over half of commonly used measures of health literacy have limited psychometric properties and often lack reporting on critical types of validity (e.g., content, construct, criterion, internal, predictive) [9, 12]. As a result, acceptable strategies are needed that address the limitations of existing HL measures, particularly in the numeracy related dimensions [9].

The S-TOFHLA is the most frequently used measure of HL, used in over half of all published papers measuring HL [9, 11]. However, it measures reading fluency, leaving out key domains in HL [8–10, 23], and is often not feasible to use in clinical settings due to limited time and resources for administering and scoring the measure [24]. Prior research has questioned established S-TOFHLA scoring and categories [9, 25–31]. Thus, the purpose of this study was to look at the performance of the S-TOFHLA in identifying those with limited numerical HL when compared to a subjective and an objective numerical HL measure.

## Methods

### Study design

This study was part of a randomized controlled trial (clinicaltrials.gov: NCT02151032) designed to investigate the use of decision aids in colorectal cancer screening. Eligible participants were English-speaking, aged 45–75 years, and had no history of colorectal cancer. Participants were recruited in person between November 2012 and January 2013 in the Greater Houston Metropolitan area from: [1] a large academic cancer center and [2] a nonprofit community foodbank, to engage those with varying HL levels.

After providing written informed consent, participants completed a battery of questionnaires, including a measure of demographic characteristics and three measures that assess HL related competencies: the S-TOFHLA, the Subjective Numeracy Scale (SNS), and the Graphical Literacy Measure (GL). Questionnaires were paper-based and completed in-person. The research assistant was present during the completion of the questionnaires and answered questions as needed. We included these four HL related measures because they represent a breadth of HL related constructs that may be of interest to researchers and of importance in clinical settings. The cut-points used to categorize HL levels are described below. These cut-points were primarily based on the S-TOFHLA since that is the most commonly used HL measure. This study was approved by the Institutional Review Board of the sponsor institution.

### Measures

#### Demographic characteristics

Participants reported sociodemographic characteristics, such as age, gender, race/ethnicity, education level, and self-rated general health status (5-point Likert scale with scores ranging from 0 = poor to 4 = excellent).

#### Short test of functional health literacy in adults (S-TOFHLA)

The S-TOFHLA is a short version of the Test of Functional Health Literacy in Adults (Table 1) [10, 32]. This objective measure was designed to evaluate general HL by assessing individuals’ ability to read and understand health-related information [9, 10, 31]. For this investigation, we used the 36-item reading comprehension subscale, which is used in approximately 71% of papers using the S-TOFHLA [9–11]. We used the 36-item version of the S-TOFHLA because of its wide acceptance and use in HL research [9–11]. Reading passages are written at 4th and 10th grade levels, and using a modified Cloze procedure, the fifth and seventh words are removed, tasking the reader to choose the best response from four choices [10, 32].

**Table 1:** Health Literacy Measures and Cut-points

Measure	Scores	Cut-points	Rationale for chosen cut-point*
Short Test of Functional Health Literacy in Adults (S-TOFHLA)			
S-TOFHLA Total Score	0–36 Range	≤16 Low 17–22 Marginal ≥23 Adequate	The S-TOFHLA has a 7 min time limit and scores range from 0 to 36 with items worth 1 point each [10]. Based on their scores, participants are classified as having “inadequate” (0–16), “marginal” [17–22], or “adequate” [23–36] HL [10, 32]. The S-TOFHLA has good validity when compared to the full Test of Functional Health Literacy (TOFHLA) and internal consistency (Cronbach’s alpha = 0.97), yet its correlation with the Rapid Estimate of Adult Literacy in Medicine (REALM) is unclear [10, 54]
Subjective Numeracy Scale (SNS)			
SNS Total Score	1–6 Mean	≤4 Low > 4 High	SNS cores range from 1 to 6 and we used the average SNS total score for analysis [17, 21, 33]. There is no established optimal cut-point for the SNS. Various methods have been used [27, 34–36]. We selected to use the median split score of 4 (median score on total and subscales) to be the cut-point for this investigation. This cut-point was chosen to compare those who are scoring above the median score to those who score below. The SNS is a reliable (Cronbach’s alpha = 0.82) and valid numeracy measure when compared to Objective Numeracy Scale items (*r* = 0.63–0.68) [17, 21].
SNS Ability Subscale	1–6 Mean	≤4 Low > 4 High	
SNS Preference Subscale	1–6 Mean	≤4 Low > 4 High	
Graphical Literacy Measure (GL)			
GL Total Score	0–13 Range	≤6 Low > 6 High	Each item is worth 1 point and scores range from 0 to 13, with higher scores suggesting higher graphical literacy [37]. There is no established optimal cut-point for the GL. The median cut-point for the total score and for the subscales (levels) was chosen to compare those who have some difficulty with graphical literacy (low) to those who do not have less difficulty with graphical literacy (high) [37, 43–46]. The GL is an adequately reliable (Cronbach’s alpha = 0.79) and valid (Construct validity = 0.54; Convergent validity = 0.50) measure of graphical literacy [37].
GL 1, Reading	0–4 Range	≤3 Low > 3 High	
GL 2, Between	0–4 Range	≤1 Low > 1 High	
GL 3, Beyond	0–5 Range	≤2 Low > 2 High	

*References reported in the table are the sources used for the cut-points

#### Subjective numeracy scale (SNS)

Numeracy is a vital element of HL and is a priority for optimizing risk communication [13, 17]. The SNS is a self-report measure of one’s subjective ability to execute math related tasks and preferences for numbers versus prose [17, 21]. It does not contain math questions and there are no correct or incorrect answers [17, 21]. The SNS contains eight items in two 4-item subscales: Ability and Preference [17, 21]. Response options are on a 6-point Likert scale and scores range from 1 to 6 [17, 21]. The average of each participant’s responses to all eight items is calculated to create their subjective numeracy score, and higher scores indicating higher subjective numeracy [17, 21]. Average scores are also calculated for the Ability and Preference subscales [33]. Since there is no universal SNS score cut-point to identify those with limited HL, we opted to use a median split to categorize participants as having either high or low HL for the total score and for each subscale [27, 34–36].

#### Graphical literacy measure (GL)

The GL is a 13-item objective measure that assesses how individuals understand graphically-presented quantitative information [37]. Understanding graphical information is an important dimension of numeracy [38]. Thus, quantitative and graphically-presented information is a critical component of HL and making health decisions [39–41]. Using graphical images, the GL assesses abilities related to graph comprehension by increasing level of difficulty: (GL 1) “the ability to read the data,” or “find specific information in the graph,” (GL 2) “the ability to read between the data,” or “find relationships in the data as shown on the graph,” and (GL 3) “the ability to read beyond the data,” or “make inferences and predictions from the data” [37, 42]. For example, one line graph shows years on the x-axis and percent of people with a fictional disease, “Adeolitis,” on the y-axis, with three questions: [1] “Approximately what percentage of people had Adeolitis in the year 2000?,” [2] “When was the increase in the percentage of people with Adeolitis higher?,” and [3] “According to your best guess, what will the percentage of people with Adeolitis be in the year 2010?” [37] Because no verified optimal score cut-point exists, scores were categorized into low and high graphical literacy based on a median split [37, 43–46].

### Description of health literacy measure cut-points

As reported in the previous section, no optimal score cut-points exist for the SNS and the GL. Thus, we used three complementary scoring strategies. First, we used the median score from the total score to identify low and high literacy groups, based on a median split. This approach is justified based on the study setting, where more than half of the participants came from a community-based organization serving underserved groups. Second, a more conservative threshold was used, where the lowest quartile of participants from the total scores were classified as having low HL, an approach common in educational literature [47, 48]. We would expect few of these participants to score within the “adequate” range on the S-TOFHLA. Third, we used the population level estimate (36th percentile) for American adults who have basic or below HL, from the 2003 National Assessment of Adult Literacy (NAAL) [4].

### Statistical analysis

Demographic characteristics, frequencies, and descriptive statistics were generated to summarize our participant population using IBM SPSS Version 23. The three S-TOFHLA HL categories (“inadequate”, “marginal”, and “adequate”) were used for scoring. For the other HL measures, we categorized the variables into high and low categories (Table 1). These categories were based on existing score cut-points or using the median split. For categorical variables, single proportion confidence intervals were generated to assess the proportion of the S-TOFHLA score levels and the other HL measures. Pearson’s Correlations were used to assess the direction and strength of correlation between total scores on the HL measures (small ≥0.10; moderate ≥0.30; large ≥0.50) [49].

## Results

### Participants

One hundred eighty-nine individuals enrolled in the study (67 from the cancer prevention center and 122 from the foodbank) and completed the questionnaires; however, two participants were excluded from the analyses due to missing data, for a total of 187 participants (Table 2). One hundred eighty-seven participants completed the 36-item S-TOFHLA, but one participants did not complete all other HL measures, which is noted in the tables. Participants’ mean age was 58 years. Over two-thirds (70%) of participants identified as Black or African American and about one-third (37%) reported some college or trade school. More than two-thirds (71%) reported good, very good, or excellent health.

**Table 2:** Demographic Characteristics.

Demographic Characteristic	Total (*n *= 187) n (%)
Age in years, Mean (SD)	58 (8.1)
45-59	110 (58.8)
60-75	77 (41.2)
Sex (female)	118 (63.1)
Race/ethnicity	
Black/African American	131 (70.1)
Hispanic/Latino	7 (3.7)
White	38 (20.3)
Mixed Race/Other	11 (5.9)
Education	
Less than high school graduate	36 (19.5)
High school graduate	49 (26.5)
Some college/trade school	68 (36.8)
College degree	32 (17.3)
Health Status	
Excellent	11 (5.9)
Very good	40 (21.4)
Good	81 (43.3)
Fair	43 (21.4)
Poor	12 (5.9)

### Health literacy measures

**Fig. 1:**
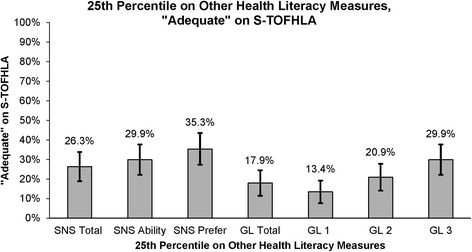
Percentage of S-TOFHLA “Adequate” Participants who Scored in Lower 25th Percentile on Other HL Measures

**Fig. 2:**
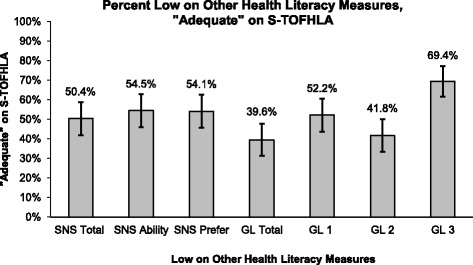
Percentage of S-TOFHLA “Adequate” Participants who Scored Low on Other Health Literacy Measures

Almost three-quarters of participants (71.7%; *n* = 134) scored “adequate” on the S-TOFHLA, while only 10.7% (*n* = 20) scored in the “marginal” category, and 17.6% (*n* = 33) scored “inadequate” (Table 3). The three strategies (median, 25th percentile, 36th percentile) to identify optimal thresholds for determining low HL for the SNS and GL reinforced each other and, therefore, we report the median and lowest 25th percentile scores to describe the results (Figs. 1 and 2). Notably, of those who scored “adequate” on the S-TOFHLA, about half scored low on SNS measures and over one third scored low on GL measures (Fig. 2).

**Table 3:** Health Literacy Measure Scores^*^

Health Literacy Measure Scores (*n* = 187)	n (%)
S-TOFHLA	
Total Score (Median (Mean [SD])	31 (26.8 [10.0])
Inadequate	33 (17.6)
Marginal	20 (10.7)
Adequate	134 (71.7)
Subjective Numeracy (SNS**)**	
SNS Total Score^†^ (Median (Mean [SD])	4 (3.9 [1.0])
Low (≤4)	100 (53.8)
High (> 4)	86 (46.2)
SNS Ability Subscale (Median (Mean [SD])	4 (3.8 [1.3])
Low (≤4)	108 (57.8)
High (> 4)	79 (42.2)
SNS Preference Subscale^†^ (Median (Mean [SD])	4 (4.1 [1.1])
Low (≤4)	101 (54.3)
High (> 4)	85 (45.7)
Graphical Literacy (GL)	
GL Total Score (Median (Mean [SD])	6 (6 [3.3])
Low (≤6)	94 (50.3)
High (> 6)	93 (49.7)
GL 1, reading the data (Median (Mean [SD])	3 (2.6 [1.5])
Low (≤2)	114 (61.0)
High (> 2)	73 (39.0)
GL 2, reading between the data (Median (Mean [SD])	1 (1.6 [1.4])
Low (≤1)	94 (50.3)
High (> 1)	93 (49.7)
GL 3, reading beyond the data (Median (Mean [SD])	2 (1.7 [1.1])
Low (≤2)	141 (75.4)
High (> 2)	46 (24.6)

^*^Numbers are shown as n and percentages unless noted otherwise

^†^*n* = 186

Of those who scored “adequate” on the S-TOFHLA, 26% (*n* = 35) of participants were in the bottom 25th percentile of the SNS Total score. Moreover, for the Ability and Preference Subscales, 30% (*n* = 40) and 35% (*n* = 47) of participants were categorized as “adequate” by the S-TOFHLA but in the bottom 25th percentile of their respective scale scores.

Forty percent (*n* = 53) of those classified as “adequate” on the S-TOFHLA were low on GL Total score. Furthermore, 18% (*n* = 24) of those scoring in the bottom 25th percentile on GL Total, scored “adequate” on the S-TOFHLA. For the GL Subscales, 13% (*n* = 18) of those classified as “adequate” on the S-TOFHLA were in the bottom 25th percentile of the GL1 Subscale. For the GL2 subscale, 21% (*n* = 28) of those classified as “adequate” on the S-TOFHLA were in the bottom 25th percentile. Lastly, 30% (*n* = 40) were classified as “adequate” on the S-TOFHLA and in the bottom 25th percentile on the GL3 Subscale.

Correlations between S-TOFHLA scores and SNS Total (*r* = 0.22, *n* = 186, *p* = 0.01; Table 4) and SNS Ability scores (*r* = 0.23, *n* = 187, p = 0.01). The correlation between the S-TOFHLA and SNS Prefer scores was not significant (*r* = 0.14, *n* = 186, *p* ≤ 0.06). The S-TOFHLA and GL Total and GL 1 scores showed large, positive correlations (*r* = 0.53, *n* = 187, *p* ≤ 0.01 and *r* = 0.52, *n* = 187, p ≤ 0.01, respectively). The correlation for the S-TOFHLA and GL3 was moderate in magnitude (*r* = 0.37, *n* = 187, *p* ≤ 0.01).

**Table 4:** Pearson Correlations Between Health Literacy Measures (*n* = 187 [unless otherwise noted])

	S-TOFHLA	SNS Total^b^	SNS Ability	SNS Prefer^b^	GL Total	GL 1	GL 2
SNS Total^b^	0.22^a^						
SNS Ability	0.23^a^	0.89^a^					
SNS Prefer^b^	0.14	0.83^a^	0.47^a^				
GL Total	0.53^a^	0.43^a^	0.41^a^	0.33^a^			
GL 1	0.52^a^	0.35^a^	0.34^a^	0.26^a^	0.91^a^		
GL 2	0.43^a^	0.40^a^	0.38^a^	0.31^a^	0.88^a^	0.73^a^	
GL 3	0.37^a^	0.34^a^	0.33^a^	0.26^a^	0.73^a^	0.50^a^	0.44^a^

^a^Correlation is significant at the 0.01 level (2-tailed)

^b^*n* = 186

## Discussion

This study raises concerns about the 36-item S-TOFHLA, a commonly used measure that has been used to identify individuals with low HL, in identifying individuals with limited numeracy. Results indicated that a large proportion of participants whose scores characterize them with “adequate” HL based on the S-TOFHLA scored low on measures of individuals’ ability to understand and interpret quantitative information.

Our results suggest that individuals categorized as having low HL on quantitative HL measures will be misclassified as having “adequate” HL with the S-TOFHLA. This is critical, as individuals with HL difficulties are at-risk for slipping through the cracks and may not receive the numerical support they need if they are screened with the S-TOFHLA. The S-TOFHLA only assesses limited aspects of HL, and yet, it persists as the most commonly-used HL measure in both research and clinical contexts [9, 11]. The current results support previous findings that participants are over-classified with “adequate” HL on the S-TOFHLA when compared to other HL measures [9, 11, 25–30, 50]. Moreover, our findings build on existing literature by adding evidence for the notable numeracy and graphical deficits of the widely used 36-item S-TOFHLA, challenging the utility of the S-TOFHLA and its use as a general HL measure.

The SNS and GL Total and Subscale score findings highlight the deficits of the S-TOFHLA for assessing basic and advanced numeracy skills, such as understanding risk, probabilities, percentages, and frequencies. While the 36-item S-TOFHLA was not designed specifically to assess numeracy, it is being used to assess general HL, of which numeracy is a critical component. Additionally, objective and subjective measures may capture different skills associated with HL and using both types of questions may be needed to reduce participant burden without compromising clinical utility. Correlations between the S-TOFHLA and the SNS were small to moderate, while correlations between the S-TOFHLA and the GL scales were moderate to large. The latter correlations between the S-TOFHLA and the GL scales may be due to both being objective measures. Despite these associations, the S-TOFHLA still misclassified many individuals based on the numeracy scores. Our findings question the broad acceptance and use of the S-TOFHLA as a universal measure of HL. A more systematic approach that provides supports for those who have deficits in HL may be a better intervention strategy rather than over-relying on limited, individual HL measures.^51–54^

Our findings add to the understanding of challenges associated with HL measurement. In order to make informed choices, patients must understand the likelihood of achieving a benefit or a harm from a treatment. Approaches that identify those with limited HL and numeracy are needed to ensure that patients receive support (if needed) to engage in these types of decisions. HL is a complex construct, and existing literature identifies and describes multiple ways of measuring it. For example, Duell et al. identified three levels for HL measurement: basic, communicative/interactive, and critical HL [9]. These levels are similar to the three GL Levels: reading the data, reading between the data, and reading beyond the data [37, 42]. In the current study, over half of those who scored “adequate” on the S-TOFHLA scored low on the GL1 subscale (reading the data). Additionally, about two-thirds of those who scored “adequate” on the S-TOFHLA scored low on the GL3 subscale (reading beyond the data). This can be observed in the correlations between the S-TOFHLA score and the GL subscales scores decrease as the GL level increases, suggesting that the S-TOFHLA may not adequately capture these more advanced level numeracy skills. For promoting patient involvement in medical decision making, numeracy is a primary skill needed to understand risk, probabilities, percentages, frequencies and trade-offs [14, 18]. Results highlight how those scoring “adequate” on the S-TOFHLA lack not only the advanced skills, but the basic HL skills needed to function in healthcare settings which may inhibit patient engagement in medical decision making.

There are various approaches to help address the challenges associated with measuring HL. First, the assumption that a single HL measure is adequate may not be the case. The HL measures included in our investigation show the need for capturing the complex skills that make-up HL. While previous studies have provided evidence to push back against commonly-used measures, such as the S-TOFHLA and REALM, a continued effort to challenge the expected use of one of these tools as a way to definitively identify those with low HL is needed [11, 50]. Furthermore, simply challenging the existing score cut-points employed by the S-TOFHLA may not be enough to identify those with limited HL skills as our findings show the discordance compared to objective and subjective numerical HL measures using multiple score cut-points. Second, there is a need for the development of a feasible strategy to capture patients’ ability to interpret and apply quantitative information in clinical and research settings. Developing strategies that incorporate subjective and objective factors critical to assessing HL, such as graphical literacy, culture, physiological condition, and relevance to disease type, are to be considered in updated measurement strategies [23, 51]. Third, is the priority for incorporating HL principles and strategies to support patient-centered care [51–53]. Strategies such as narratives, engaging storytelling and other visual supports may reduce patient burden and promote engagement for those with both high and low HL.

This research study has potential limitations. This study was conducted in a large urban area using a convenience sample. Thus, the sample is diverse and matches the makeup of large urban centers, but rural patients may have not been well represented which may impact generalizability. Second, we used the 36-item S-TOFHLA measure, which does not assess numeracy. Although this measure is broadly accepted [10], including the additional four numeracy items may have provided more detailed numeracy information. We used cut-points to categorize HL levels, which is consistent with research and clinical use of the S-TOFHLA and enabled comparisons between measures. Optimal score cut-points did not exist for the SNS and GL. To address this limitation, we used a median split approach, and more generous score cut-points of the 25th and 36th percentiles. With this strategy, we were able to present different measure score cut-points and compare them to the S-TOFHLA categories.

## Conclusions

The S-TOFHLA is limited in its measure of HL. Though widely used, those with inadequate HL skills may be over-classified as having “adequate” HL, based on their S-TOFHLA scores. Use of HL measures that include numeracy, graphical, and preference questions is essential when considering measuring HL. Existing numeracy or graphical measures may be too cumbersome for the clinical setting but have higher utility when categorizing those with high and low numerical skills, which may be of particular interest to those developing decision support tools. Developing a brief tool with both subjective and objective quantitative questions may augment HL numeracy measurement.

## Abbreviations

GL: Graphical Literacy Measure
HL: Health Literacy
NAAL: National Assessment of Adult Literacy
SNS: Subjective Numeracy Scale
S-TOFHLA: Short Test of Functional Health Literacy in Adults
TOFHLA: Test of Functional Health Literacy in Adults
US: United States
